# The power of siblings and caregivers: under-explored types of social support among children affected by HIV and AIDS

**DOI:** 10.1080/09540121.2016.1178942

**Published:** 2016-07-08

**Authors:** Melissa Sharer, Lucie Cluver, Joseph J. Shields, Frederick Ahearn

**Affiliations:** ^a^ John Snow Research & Training Institute, Arlington, VA, USA; ^b^ The National Catholic School of Social Service, The Catholic University of America, Washington, DC, USA; ^c^ Department of Social Policy & Intervention, University of Oxford, Oxford, UK; ^d^ Department of Psychiatry and Mental Health, University of Cape Town, Cape Town, South Africa

**Keywords:** Children, HIV, family, mental health, social support

## Abstract

Children affected by HIV and AIDS have significantly higher rates of mental health problems than unaffected children. There is a need for research to examine how social support functions as a source of resiliency for children in high HIV-prevalence settings such as South Africa. The purpose of this research was to explore how family social support relates to depression, anxiety, and post-traumatic stress (PTS). Using the ecological model as a frame, data were drawn from a 2011 cross-sectional study of 1380 children classified as either orphaned by AIDS and/or living with an AIDS sick family member. The children were from high-poverty, high HIV-prevalent rural and urban communities in South Africa. Social support was analyzed in depth by examining the source (e.g. caregiver, sibling) and the type (e.g. emotional, instrumental, quality). These variables were entered into multiple regression analyses to estimate the most parsimonious regression models to show the relationships between social support and depression, anxiety, and PTS symptoms among the children. Siblings emerged as the most consistent source of social support on mental health. Overall caregiver and sibling support explained 13% variance in depression, 12% in anxiety, and 11% in PTS. Emotional support was the most frequent type of social support associated with mental health in all regression models, with higher levels of quality and instrumental support having the strongest relation to positive mental health outcomes. Although instrumental and quality support from siblings were related to positive mental health, unexpectedly, the higher the level of emotional support received from a sibling resulted in the child reporting more symptoms of depression, anxiety, and PTS. The opposite was true for emotional support provided via caregivers, higher levels of this support was related to lower levels of all mental health symptoms. Sex was significant in all regressions, indicating the presence of moderation.

In South Africa, an estimated 20% of children (2.5 million) have lost at least one parent due to AIDS (DeSilva et al., 2012). The number of children affected by HIV is even higher (Sherr et al., 2014) and these children face additional psychological and health burdens (Betancourt, Meyers-Ohki, Charrow, & Hansen, 2013; Short & Goldberg, 2015). Many are affected at an early age when parental, community guidance, and socialization are most desirable (Atwine, Cantor-Graae, & Bajunirwe, 2005). The loss experienced by these children can impact their mental health throughout their lifetime. Researchers are only just beginning to understand the numerous pathways and interventions which may promote positive wellbeing and thus improve the resilience of these children (Betancourt et al., 2013; Desmond et al., 2014; Sherr et al., 2014) (Figure 1).

**Figure 1.**
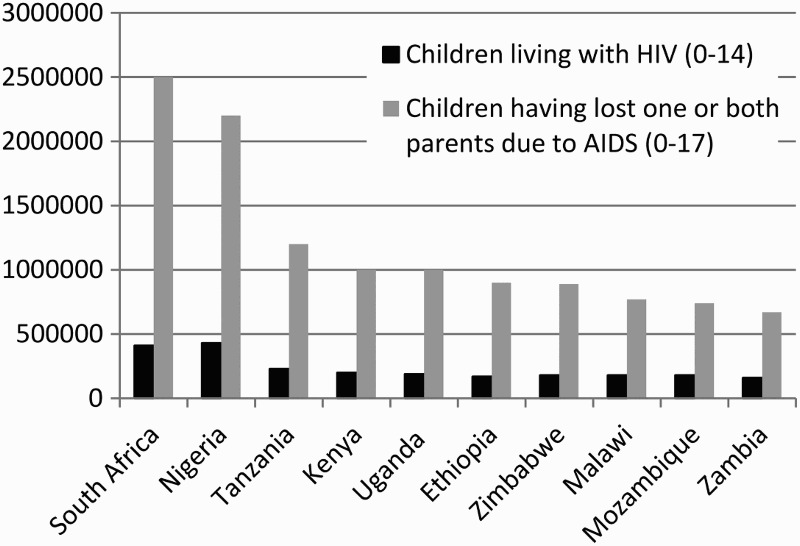
Children living with HIV (0–14) and children having lost one or both parents due to AIDS (0–17) in the most affected countries in Sub-Saharan Africa by number (WHO, 2015b).

Bronfenbrenner’s ecological theory can frame an examination of the linkages between these children within the context of their families (Fraser, 2004). Acknowledging that a child does not develop in isolation, ecological theory examines the layers surrounding a child (Stein et al., 2014). Ecological theory frames this study by aiming to understand how existing relationships in a child’s microsystem (caregivers and siblings) may illustrate the types of social support that improve the child’s psychological wellbeing (Betancourt et al., 2013; Cluver, Operario, & Gardner, 2009). This research’s purpose was to explore how the source (caregivers and siblings) and types (emotional, instrumental, quality) of social support relate to symptoms of depression, anxiety, and post-traumatic stress (PTS).

## Mental health

Strong evidence suggests that children orphaned due to AIDS have much higher rates of mental and psychological stress when compared to other orphans and youth not orphaned (Cluver, Orkin, Gardner, & Boyes, 2012; Lee, 2012; Sherr et al., 2014). However, recent studies indicate children who are not yet orphaned, but living with an AIDS ill caregiver may also experience higher levels of psychological distress (Eloff et al., 2014). Many of these children have not yet been told of their caregiver’s HIV status, and disclosure within families remains a struggle, despite the health and psychological benefits associated with the process (Cheney, 2015). The vulnerabilities associated with being orphaned due to AIDS starts long before a child’s parent dies, with children witnessing the devastating affects within their own home (Skovdal & Daniel, 2012). Recent WHO (2015a) recommendations state that all those living with HIV, regardless of CD4 count, be initiated on ART immediately. As this occurs, more children will be living with a HIV-positive family member, resulting in new and unanticipated challenges and opportunities (Short & Goldberg, 2015). Many of these affected children may experience increased stigma and psychological distress as their caregiver’s symptoms emerge, thus impacting the child’s functioning (Cluver et al., 2012; Eloff et al., 2014; Short & Goldberg, 2015).

## Social support types and sources

Social support may have protective factors related to positive mental health among youth affected by HIV and AIDS (Cheng et al., 2014; Okawa et al., 2011). Social support is a form of social capital documenting the existence of relationships, networks, and connections between people (Verudin, Smid, Wind, & Scholte, 2014). These relationships (sources) and types of social support remain a nebulous and under-researched concept among children affected by HIV and AIDS (Wang et al., 2012).

Most research focuses on the interaction between perceived quality of support and psychological wellbeing (Cheng et al., 2014; DeSilva et al., 2012; Okawa et al., 2011; Wang et al., 2012). Perception of quality is critical for a child to feel supported and connected. Other, under-researched types of social support include emotional and instrumental. Emotional support is an individual’s ability to show empathy, provide love, trust, and care (Cummings et al., 2014). Instrumental support refers to the provision of money/goods (Casale, Wild, Cluver, & Kuo, 2014).

Families in South Africa are often wide ranging and hard to define, but they commonly enable protection and nourishment, even under extreme circumstances (Cook & White (Xelimuxw), 2006; Evans, 2005). Families are a primary safety net for children and in many HIV-impacted areas, they may be emotionally and financially stretched (Heymann & Kidman, 2009; Richter et al., 2009). Children have multiple sources of support; however, there is limited research on how sibling support relates to a child’s wellbeing (Cheng et al., 2014). Siblings are a critical relationship for any child although limited research exists describing the supportive nature of these relationships in the context of parental HIV and AIDS. Research from Kenya and China found siblings support to be protective for mental health outcomes among children orphaned by AIDS (Hong et al., 2010; Okawa et al., 2011). This study examines the specific sources and types of social support to increase the understanding of how social support relates to the mental health of children affected by HIV/AIDS.

## Methodology

The current study uses 2011–2012 cross-sectional longitudinal data from the Young Carers Project in South Africa. Data were collected from urban and rural districts in Mpumalanga and Western Cape Provinces. Census enumeration areas were randomly selected in each province, and within these all households with a child aged between 10 and 17 were asked to join (2.5% refusal) (Cluver, Orkin, Boyes, & Sherr, 2014). Youth provided verbal assent and written consent. Caregivers provided additional written consent (Meinck, 2014).

### Study population

Out of the 3516 participants, 1380 were classified as children affected by HIV and AIDS (mean age 14.8, median age 15, ranging from ages 10 to 20). This extremely vulnerable group was this research’s primary focus. To determine if orphan-hood was due to AIDS, the research team used Verbal Autopsy method, as South African death certificates do not state if the cause of death was due to HIV/AIDS-related complications. Verbal Autopsy, validated in South Africa (Hosegood, Vanneste, & Timaeus, 2004), was also used to determine AIDS-illness among all household members, with children reporting illness for at least two weeks. Similar to validating death due to AIDS, household AIDS-illness was conservatively determined by the presence of three or more AIDS-defining symptoms (Meinck, 2014). When diagnoses were unclear, symptoms were reviewed independently by medical professionals. Child report has successfully shown good reliability in previous studies (Boyes, Mason, & Cluver, 2013).

### Mental health measures

Three scales examined depression, anxiety, and PTS symptoms, the study’s three dependent variables. All scales were totaled and cutoffs were avoided as there are no clinical cutoffs validated in Africa (Cluver, Kganaka, Boyes, & Park, 2012). The survey used the 10 question short-form of the Child Depression Inventory (Kovacs, 1992). This scale, with an internal consistency of .75 (alpha), has been used frequently in South Africa (Boyes & Cluver, 2013). To measure anxiety, a reduced version of the Children’s Manifest Anxiety Scale-Revised was employed, and has been validated among youth in South Africa (Boyes & Cluver, 2013). Reliability was strong (.83). PTS was measured by the Child PTSD Checklist (Amaya-Jackson, Newman, & Lipschitz, 2000). This Checklist had an internal consistency of .92 and has been validated in South Africa (Boyes, Cluver, & Gardner, 2012).

### Social support measures

The Social Support Scale (Seidman et al., 1995) identified two sources of social support at the family level, caregivers and siblings (independent variables). This scale was adapted replacing “mother” and “father” with “caregiver” to allow for the diversity that exists in the context of South Africa (Cluver et al., 2009). Three types of support were specified, emotional, instrumental, and perceived quality. Both caregiver and sibling were listed as distinctly separate sources. Children reported the type of support they received from “people in your life” covering an unspecified time period. Emotional support (“this person is helpful when I have a personal problem”), instrumental support (“this person is helpful when I need money and other things”), and perceived quality of support (“I have fun with this person”) were measured using a categorical scale (1 = not at all, 2 = sort of, and 3 = very).

### Control variables

Socio-demographic control variables included age, sex, rural/urban, household size, caregiver relation (kin/non-kin), and economic status proxies (formal/informal household structure and in home/out of home water source) (Filmer & Pritchett, 2001). The child had multiple options to select for their primary caregiver, resulting in more than 20 descriptors, which were analyzed in numerous configurations, and ultimately dichotomized into kin/non-kin caregivers.

### Human subject concerns

This research received ethical approval from Oxford University, University of Cape Town, and the University of KwaZulu-Natal, and The Catholic University of America’s Committee for the Protection of Human Subjects.

## Data analysis

All analyses were run using SPSS, frequency distributions and bivariate correlations were assessed on all variables. Multiple regression analyses (block method) were used to examine the effects related to social support source and type as reported by 1380 youth while controlling for seven variables. No correlations above .60 were observed indicating the absence of multicollinearity (Abu Bader, 2010).

## Results

In order to examine differences by sex, we examined background characteristics for boys compared to girls. There were no significant demographical differences between the two groups of children. For the mental health variables, girls exhibited a significantly higher means for depressive, anxiety, and PTS symptoms compared to boys (Table 1).

**Table 1.** Description of children affected by HIV and AIDS.

	Girls (*N* = 830)	Boys (*N* = 550)	*λ*^2^ value *p* value	*t*-test value *p* value
Age, *M* (SD)	14.91 (2.22)	14.70 (1.97)		−.98 *p* = .33
Household size, *M* (SD)	6.06 (2.09)	5.95 (1.97)		−1.80 *p* = .08
Rural, *N* (%)	438 (52.8%)	271 (49.3%)	1.98 *p* = .16	
Water in home, *N* (%)	420 (50.6%)	265 (48.2%)	1.72 *p* = .42	
Household brick/concrete, *N* (%)	527 (63.5%)	372 (67.6%)	3.49 *p* = .33	
Caregiver kin, *N* (%)	777 (93.6%)	519 (94.4%)	4.9 *p* = .18	
Depressive symptoms, *M* (SD)	2.65 (2.21)	2.37 (2.40)		−2.20 *p* = .03*
Anxiety symptoms, *M* (SD)	5.30 (3.52)	4.59 (3.30)		−3.74 *p* = .00**
PTS symptoms, *M* (SD)	15.19 (12.72)	12.39 (11.33)		−4.21** p = .00

*p < .05.

**p < .001.

To better understand the variables most associated with depression, anxiety, and PTS symptoms multiple regression analyses were conducted. These analyses provided a comprehensive view of what family source and type of social support related to mental health. Sibling and caregiver support was distinctly reported; however depending on the child’s situation, there may be some undetected overlap between these two groups. As such, during analyses composite variables were created to reflect multiple sources of support (caregiver and sibling), with insignificant results. This resulted in analyzing each source separately to provide the most informative results (Table 2).

**Table 2.** Family: Regression of significant variables related to depression, anxiety, and PTS symptoms.

Variables	Depression				Anxiety				PTS			
	*B*	SE(*B*)	*β*	*p*	*B*	SE(*B*)	*β*	*p* value	*B*	SE(*B*)	*β*	*p* value
Sex	.37	.13	.08	.00**	.80	.19	.11	.00**	3.22	.68	.13	.00**
Age	.07	.03	.06	.02*	.07	.04	.05	.10	.07	.16	.01	.67
Rural	−.22	.13	−.05	.09	−.35	.20	−.05	.07	−1.95	.69	−.08	.01*
HH structure	.00	.00	.00	.93	−.01	.00	−.06	.04*	−.01	.01	−.04	.20
Water source	−.29	.14	−.07	.03*	−.19	.21	−.03	.35	.06	.73	.00	.93
HH #	−.07	.03	−.07	.02*	−.04	.05	−.03	.36	−.09	.17	−.02	.58
Caregiver kin	−.00	.00	−.02	.49	.00	.00	−.01	.80	−.02	.01	−.05	.06
*Caregiver*												
Emotional	−.55	.19	−.10	.00**	−1.11	.29	−.13	.00**	−2.66	1.03	−.09	.01*
Instrumental	−.08	.16	−.02	.60	−.44	.24	−.06	.07	−1.06	.84	−.04	.20
Quality	−.75	.23	−.11	.00**	−.45	.35	−.04	.19	−4.80	1.24	−.13	.00**
*Sibling*												
Emotional	.48	.11	.15	.00**	.51	.17	.10	.01*	2.99	.59	.17	.00**
Instrumental	−.44	.10	−.16	.00**	−.55	.15	−.14	.00**	−1.49	.51	−.10	.01*
Quality	−.91	.15	−.18	.00**	−1.34	.24	−.17	.00**	−4.30	.83	−.16	.00**
*R*^2^				.134				.126				.114
*F*				13.99				12.96				12.73

Note: HH = household.

Sex (0 = girl, 1 = boy); age (continuous); urban = 1 and rural = 2; household (HH) structure (0 = informal non-brick/cement structure and 1 = formal brick/cement structure); water source (0 = outside home water source, 1 = in home water source); household (HH) size (continuous); caregiver non-kin = 0, kin = 1.

*p < .05.

**p < .001.

### Family social support and mental health

Siblings emerged as the most consistent source of social support significantly associated with mental health. Specifically, when siblings provided instrumental and high quality support, children were less likely to experience symptoms of anxiety, depression, and PTS. Unexpectedly, the higher the level of emotional support received from a sibling, the higher number of mental health symptoms the child reported. The relationship between emotional and mental health outcomes was anomalous. Higher levels of emotional support from caregivers and the quality of that support was related to lower number of symptoms for all three mental health outcomes. However, there was no significance between instrumental support from caregivers and mental health.

For depression, four control variables were all significantly associated with a higher number of depression symptoms. Being a girl (*β* = .08, *p* < .001), being older (*β* = .06 *p* < .05), having no in-home water source (*β* = −.07, *p* < .05), and having less people in your home (*β* = −.07, *p* < .05) were related to higher number of depression symptoms. Caregiver emotional support and the higher the perceived quality of support resulted in less symptoms of depression among the children (emotional with *β* = −.10, *p* < .001, quality with *β* = −.11, *p* < .001). Siblings who provided instrumental and quality support resulted in lower number of depression symptoms among the children (instrumental (*β* = −.16, *p* < .001, and quality with *β* = −.18, *p* < .001). Emotional was reversed, with children receiving emotional support from their siblings reporting higher numbers of symptoms (emotional with *β* = .15, *p* < .001). The final depression regression model included nine significant variables (*F*(9, 1175) = 19.80, *p* < .001) which accounted for 13.4% of the variance in depression symptoms (*R* ^2^ = .134).

For anxiety, girls (*β* = .11, *p* < .001) and children living in informal housing (*β* = −.06 *p* < .05) were significantly more likely to report anxiety symptoms. Children who reported caregiver emotional support had less anxiety symptoms (emotional with *β* = −.13, *p* < .001). Siblings who provided instrumental and quality support resulted in lower number of anxiety symptoms among the children (instrumental *β* = −.14, *p* < .001, quality with *β* = −.17, *p* < .001). Once again emotional was reversed, with children receiving sibling emotional support experiencing higher numbers of symptoms (emotional with *β* = .10, *p* < .05). The final regression model for anxiety included six significant variables (*F*(6, 1182) = 25.25, *p* < .001), which accounted for 12.6% of the variance in anxiety symptoms (*R* ^2^ = .126).

Girls (*β* = .13, *p* < .001) and children living in an urban setting (*β* = −.08 *p* < .05) predicted higher number of PTS symptoms in the final regression model. The final model also included two type of caregiver support (emotional with *β* = −.09, *p* < .05, and quality with *β* = −.13, *p* < .001), and three types of sibling support (emotional *β* = .17, *p* < .001, instrumental *β* = −.10, *p* < .05, and quality *β* = −.16, *p* < .001). The same pattern held true, all support except emotional support from siblings, resulted in less negative symptoms of mental health. The final regression model included six significant variables, which accounted for 11.4% of the variance in PTS symptoms (*R* ^2^ = .114).

## Discussion

Examining multiple types of familial social support in greater detail than any known study to date; this research contributes to a deeper understanding about the construct of social support. New information emerged underscoring the significance of sibling social support. These results reinforce the importance of a child’s perception of quality, as if he/she was happy with the support received s/he was more likely to report positive mental health. When a child received instrumental support from their siblings, they had lower number of depression, anxiety, and PTS symptoms; however, caregiver instrumental support had no relationship. To understand this finding more deeply, instrumental support was further analyzed in relation to the economic proxy variables (in-home water source and brick/concrete house) with mixed results. No significance emerged from caregiver instrumental support; however, sibling instrumental support exhibited contradictory relationships. Children who lived in permanent housing were significantly less likely to receive sibling instrumental support, and those with an in-home water source were significantly more likely to receive instrumental support from their siblings. Future research should focus on how instrumental support may relate to a child’s economic status.

Emotional social support emerged with mixed results. Caregiver emotional support was related to lower number of depression, anxiety, and PTS symptoms. These findings were reversed for sibling emotional support, with higher levels of emotional support related to higher levels of depression, anxiety, and PTS symptoms. This was unanticipated, and in attempts to determine the deeper relationship between emotional support from siblings and negative mental health, this finding was explored in multiple ways to see if it could be explained via interaction affects with variables such as sex and orphaned status, with no significant findings. As siblings are also children affected by HIV and AIDS, and there may be negative outcomes occurring from a shared sense of grief, perhaps leading to rumination and an increased focus on distress. This emotional support appears harmful, but it may be a replacement for something more harmful, as no coherent pathway was found via deeper analysis. These results require more research to more deeply understand this relationship. For the most part, psychological wellbeing was positively influenced by family social support. Additionally the regression model revealed that girls had worse mental health outcomes than boys; future research should also examine the potential moderator effects of sex.

### Practice implications

These results lead to an improved understanding of how family, and in particular siblings, provide support for children affected by HIV and AIDS. This may allow more precision when developing programs and delivering services in South Africa; however, the results may inform other programs in Sub-Saharan Africa as well. Results reinforce that family strengthening should target both caregiver and sibling relationships. Siblings, also children affected by HIV and AIDS, should be recognized as powerful sources of support. Working directly with this group and strengthening the sibling’s skills related to support provision may also increase the child’s internal and external coping mechanisms. Additionally, these data strongly indicate the need for regular mental health assessment and follow-on support for all children affected by the HIV epidemic. Establishing universal mental health screenings for all children living in high HIV-prevalence settings should be prioritized.

### Policy implications

Understanding that both caregivers *and* siblings are a critical source of social support for these children allows policy-makers to use limited funds in the most efficacious manner. An ecological frame may guide policy-maker’s efforts to better meet the needs of children and their families affected by HIV and AIDS.

### Research implications

All children in the HIV epidemic would benefit from research that uses an ecological frame, moving away from a deficit-based model to a strengths-based perspective (Masten, 2014). The current study adds to the body of knowledge and evidence around social support, mental health, and children affected by HIV and AIDS. Research should continue to be conducted among underrepresented groups, and have a family-centered focus that explores strengths among both siblings and caregivers. Additionally, intervention research should examine ways to reinforce existing familial relationships, to deepen the understanding of how activities can improve the mental health of all children and their families.

This inquiry provides more evidence on what types and sources of support were related with stronger wellbeing. Emphasizing which sources of support are most critical for the child, in addition to highlighting the most important types of social support is critical information that can lead to strengthened health and social welfare systems to ensure that all HIV-affected children receive services that strengthen their overall wellbeing (Cheng et al., 2014; Nyamukapa et al., 2010; Stein et al., 2014). With the new treatment guidance (WHO, 2015a), it is likely that more children will reside with HIV-positive family members living, which may increase risks of morbidity and mortality, inclusive of mental health problems (Short & Goldberg, 2015). This reinforces the need to ensure all combined efforts (programs, policies, and research) focus on uncovering and strengthening naturally occurring support mechanisms within families. Families remain neglected and this analysis supports the emerging recommendations that call for increased attention to the holistic needs of children affected by AIDS and their families (Short & Goldberg, 2015). Effective programs and policies should be designed to reinforce existing and develop new family support mechanisms for this growing number of children affected by HIV and AIDS.
